# Cholera Mortality during Urban Epidemic, Dar es Salaam, Tanzania, August 16, 2015–January 16, 2016^1^

**DOI:** 10.3201/eid2313.170529

**Published:** 2017-12

**Authors:** Lindsey S. McCrickard, Amani Elibariki Massay, Rupa Narra, Janneth Mghamba, Ahmed Abade Mohamed, Rogath Saika Kishimba, Loveness John Urio, Neema Rusibayamila, Grace Magembe, Muhammud Bakari, James J. Gibson, Rachel Barwick Eidex, Robert E. Quick

**Affiliations:** Centers for Disease Control and Prevention, Atlanta, Georgia, USA (L.S. McCrickard, R. Narra, R.E. Quick);; United Republic of Tanzania Ministry of Health, Community Development, Gender, Elderly, and Children, Dar es Salaam, Tanzania (A.E. Massay, J. Mghamba, R.S. Kishimba, N. Rusibayamila, M. Bakari);; Tanzania Field Epidemiology and Laboratory Training Program, Dar es Salaam (A.E. Massay, A.A. Mohamed, R.S. Kishimba, L.J. Urio, J.J. Gibson); Regional Secretariat, Dar es Salaam (G. Magembe);; US Centers for Disease Control and Prevention, Dar es Salaam (J.J. Gibson, R.B. Eidex)

**Keywords:** cholera, *Vibrio cholerae*, mortality, Tanzania, epidemic, epidemiology, surveillance, global health security, bacteria

## Abstract

In 2015, a cholera epidemic occurred in Tanzania; most cases and deaths occurred in Dar es Salaam early in the outbreak. We evaluated cholera mortality through passive surveillance, burial permits, and interviews conducted with decedents’ caretakers. Active case finding identified 101 suspected cholera deaths. Routine surveillance had captured only 48 (48%) of all cholera deaths, and burial permit assessments captured the remainder. We interviewed caregivers of 56 decedents to assess cholera management behaviors. Of 51 decedents receiving home care, 5 (10%) used oral rehydration solution after becoming ill. Caregivers reported that 51 (93%) of 55 decedents with known time of death sought care before death; 16 (29%) of 55 delayed seeking care for >6 h. Of the 33 (59%) community decedents, 20 (61%) were said to have been discharged from a health facility before death. Appropriate and early management of cholera cases can reduce the number of cholera deaths.

Cholera is an acute diarrheal illness caused by infection with the bacterium *Vibrio cholerae* (*1*). Severe cholera can be rapidly fatal; patients who do not receive appropriate treatment could die within hours (*1*). Prompt replacement of fluids and electrolytes through the use of oral rehydration solution (ORS) and intravenous fluids can prevent cholera death (*2*). With appropriate care, case-fatality rates for cholera should be <1% (*1*).

Tanzania reported an outbreak of cholera on August 15, 2015 (*3*,*4*). At that time, 6 of 8 countries bordering Tanzania were experiencing cholera outbreaks (*5*). Cholera outbreaks can spread rapidly, crossing national borders, and are a major global health security problem.

Early in the Tanzania outbreak, most cases and deaths were reported in Dar es Salaam, where 3,371 cases and 36 deaths (case-fatality rate 1.1%) had been recorded by October 31, 2015. Deaths were exclusively reported from cholera treatment centers (CTCs), but additional deaths in the community were rumored. When cholera deaths in the community were suspected, an environmental health officer was required to visit the decedent’s house, prepare a burial permit, obtain a rectal swab for culture, and assist with the disposal of the body. The burial permit included the decedent’s name, suspected cause of death, and date of death. We conducted a cholera mortality evaluation to identify unreported deaths, investigate household cholera management practices, and describe healthcare-seeking behaviors.

## The Study

We obtained a list of persons who were suspected to have died of cholera (decedents) from the CTCs and obtained the burial permits from the CTCs, referral hospitals, and municipal offices (for complete description of methods, see Technical Appendix 1. The case definition for suspected cholera death was death of a person ≥2 years of age with acute watery diarrhea with or without vomiting with illness onset after August 15, 2015, in Dar es Salaam. A confirmed cholera death was defined as death of a person ≥2 years of age whose stool was positive for *Vibrio cholerae* O1 (*6*). All suspected and confirmed cholera deaths identified from CTC reports and burial permits were included in the evaluation.

We developed survey instruments with the Open Data Kit software (https://opendatakit.org/). Written informed consent to take surveys was obtained, and then trained enumerators completed surveys with caregivers or relatives of the deceased (Technical Appendix 2). During January 19–23, 2016, these data were collected electronically on Galaxy Tablets (Samsung, Seoul, South Korea).

**Figure 1:**
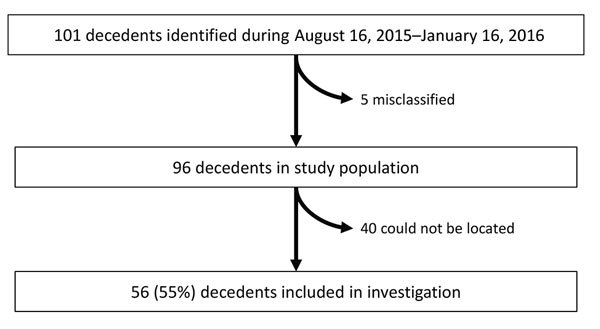
Study population for cholera mortality evaluation, Dar es Salaam, Tanzania, August 16, 2015–January 16, 2016.

During August 16, 2015–January 16, 2016, the cholera surveillance system in Dar es Salaam identified 48 cholera deaths, all reported by CTCs. These deaths included persons who died at CTCs and persons who were dead on arrival. The burial permit assessment identified an additional 53 cholera deaths for a total of 101 total deaths (Figure 1); therefore, 52% of the total deaths were not captured by the existing surveillance system. 

**Figure 2:**
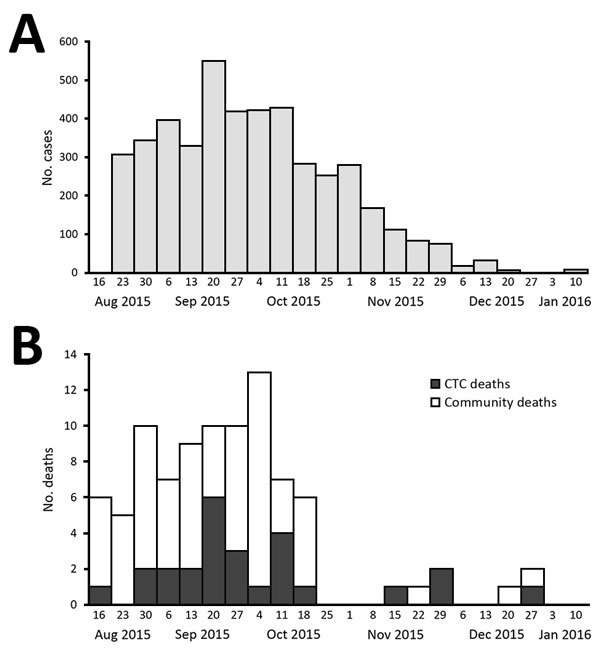
Suspected and confirmed cholera cases (A) and deaths (B) from cholera mortality evaluation and burial permit assessment, by week, Dar es Salaam, Tanzania, August 16, 2015–January 16, 2016. CTC deaths are deaths in patients >2 years of age with suspected or confirmed cholera who died following admission to a hospital or CTC. Community deaths were deaths in persons >2 years of age highly suspected of having cholera or having culture-confirmed cholera who died in the community or en route to a CTC. The date of death could not be determined for 6 decedents who were therefore excluded from the epidemic curves. CTC, cholera treatment center.

Cholera cases and deaths peaked in late September, with fewer deaths reported from November through January (Figure 2, panels A, B). The decrease in deaths coincided with a decrease in reported cholera cases. Of 101 decedents, 45 (45%) were not included in the study: for 35 (87.5%), caretakers could not be located; for 3 (7.5%), the caretakers had moved; 2 (5%) were an entire family unit with no respondent to give a survey; and 5 were misclassified (2 were <2 years of age and 3 had negative cultures with clinical signs inconsistent with cholera). Anecdotal reports suggested that many of the decedents for whom family members and caretakers could not be found were migrant workers who lived alone in single rented rooms, and the homes of others were not disclosed because of the stigma associated with cholera and local political pressure not to report.

Caretakers interviewed for this evaluation were family members (73%), landlords and neighbors (21%), employees (4%), and friends (2%) of the 56 decedents. The median age of decedents was 23 (range 2–80) years, and 32 (57%) were men or boys (Table). 

**Table:** Demographic characteristics of cholera decedents from cholera mortality evaluation, Dar es Salaam, Tanzania, August 16, 2015–January 16, 2016*

Characteristic	Total, N = 56	Confirmed, n = 39	Suspected, n = 17
Median age, y (range)†	23 (2–80)	21 (2–80)	25.5 (3–73)
Sex			
M	32 (57)	24 (62)	8 (47)
F	24 (43)	15 (38)	9 (53)
Location of death			
Health facility	22 (39)	11 (28)	11 (65)
Community	33 (59)	28 (72)	5 (29)
Unknown	1 (2)	0 (0)	1 (6)
Clinical signs/symptoms			
Vomiting	42 (75)	28 (72)	14 (82)
Diarrhea‡	43 (77)	28 (72)	15 (88)
Headache	9 (16)	7 (18)	2 (12)

*Values are no. (%) decedents except as indicated. †Age was unknown for 7 persons. ‡One respondent did not know if the patient had diarrhea. Caretakers of 2 persons with suspected cases did not report diarrhea but met the burial permit assessment case definition.

Fecal samples from 39 (70%) decedents yielded *V. cholerae*. Laboratory results from 16 (29%) decedents were not available. The location of death was the community or en route to a health facility for 33 (59%) decedents, a health facility for 22 (39%), and an unknown location for 1 (2%) (Table). Of the 51 respondents who reported that decedents received home treatment, 5 (10%) said ORS was consumed. Reasons the decedents did not take ORS at home included not knowing what ORS was (38%) and not thinking that ORS would help (33%). Of 56 decedents, 26 (46%) consumed fluids other than ORS, including water (30%), soft drinks (13%), and porridge (5%), at home before their deaths.

Of 55 decedents with a reported time of death, 44 (80%) died within 24 hours of symptom onset, and of the 51 (93%) decedents who sought care before death, 16 (31%) waited >6 h from symptom onset to seek care. All decedents were able to reach a health facility from their home within 1 hour. Of 33 decedents who died in the community or en route to a health facility, 20 (61%) had previously been discharged alive from a health facility.

## Conclusions

More than half of the records of cholera deaths in Dar es Salaam were missing from the existing surveillance system, which only captured patients who arrived at CTCs. Deaths that occurred in other treatment locations or in the community were not reported. Underreporting of deaths during cholera epidemics, a phenomenon not unique to Tanzania (*5*,*7*,*8*), poses a threat to global health security.

We identified 3 anecdotal barriers to reporting cholera deaths. One barrier was political pressure; because of the electoral campaign ongoing during the epidemic, healthcare workers might have been discouraged from reporting cholera cases (*9*,*10*). Similarly, in 2008, underreporting of cholera deaths was observed during an electoral campaign in Kenya (*8*). Another barrier was influence from local leaders; because of the stigma associated with cholera, these leaders might have wished to deny the presence of the disease in their communities and created an environment discouraging others from reporting (*11*,*12*). The third barrier was lack of communication with immigrants; some decedents reported to be migrant workers who lived alone did not have social contacts who could serve as caregivers or report the decedent’s cause of death. Similar observations have been described in another cholera mortality investigation (*13*).

This evaluation suggested that most caregivers of decedents lacked knowledge of ORS. Other studies have observed that the use and knowledge of ORS (*7*,*8*,*14*) has plateaued or declined in countries of sub-Saharan Africa and Asia since the 1990s (*15*,*16*). This decline or plateau was associated with decreased funding for diarrhea control projects, declining commercialization of ORS, and inconsistent messaging regarding homemade ORS (*16*). In addition, >30% of cholera decedents delayed seeking care by >6 h. Although other cholera mortality studies have not directly addressed the effect of delays in seeking care, several studies have identified distance to health facilities or lack of transportation as barriers to timely care in rural populations (*14*,*17*,*18*). In this urban epidemic, all decedents were able to reach a health facility within 1 hour. The failure to seek timely care was probably a matter of inadequate messaging to the public.

More than 60% of community decedents were reportedly discharged from a health facility before dying, suggesting inadequate management by health workers or premature discharge. The Tanzanian Ministry of Health initiated healthcare provider training in November 2015 to address cholera case management problems; starting around that time, cholera deaths became infrequent (Figure 2, panel B). The use of rectal swabs to confirm cholera in decedents might be a useful practice especially in the context of unexplained deaths during cholera outbreaks. 

Enhanced surveillance, cholera case management training, and robust community education focused on destigmatizing the disease, as well as encouraging persons on the margins of society to seek medical attention for cholera-like symptoms, are needed to manage cholera epidemics. These practices can help expedite outbreak detection and response, facilitate the control of cholera at its source, and prevent deaths, enhancing global health security.

Technical Appendix 1 Description of methods used to evaluate cholera mortality.

Technical Appendix 2 Survey given to caregivers of decedents.
