# Supplementary material for: Cholera Mortality during Urban Epidemic, Dar es Salaam, Tanzania, August 16, 2015–January 16, 2016
Source: Emerg Infect Dis. 2017 Dec;23(Suppl 1):S154–7. doi: 10.3201/eid2313.170529 (PMC5711300; doi:10.3201/eid2313.170529)
Supplement: Technical Appendix 1 — Description of methods used to evaluate cholera mortality. [file 17-0529-Techapp-s1.pdf]

Article DOI: https://doi.org/10.3201/eid2313.170529

Cholera Mortality during Urban Epidemic, Dar es Salaam, Tanzania, August 16, 2015–January 16, 2016

Technical Appendix

Methods

Type of Investigation

This investigation was a descriptive and comparative study of a series of cholera decedents. An attempt was made to compare differences between cholera treatment center (CTC) and community cholera deaths.

Investigation Population

The investigation population was a convenience sample of all decedents ≥2 years of age with suspected or confirmed cholera in Dar es Salaam, Tanzania, from August 16, 2015, through January 16, 2016. The investigation included all persons who died in a hospital or a CTC, in the community, or on the way to a hospital or CTC, and whose burial was assisted with environmental health officers (EHOs). All deaths caused by cholera reported in burial permits in the investigation area were included. Inclusion criteria were a) dying of suspected or confirmed cholera in Dar es Salaam; b) being ≥2 years of age; c) dying during August 15, 2015–January 16, 2016; and d) having a burial assisted by the municipal burial team from the relevant district in Dar es Salaam. Data from decedents without burial permits and decedents whose caretakers did not consent to the survey were excluded.

Investigation Area

The following hospitals and CTCs in Dar es Salaam participated: Amana Hospital, Mwanyamala Hospital, Temeke CTC at Temeke Hospital, Vijibweni CTC, Mburahati CTC at Mburahati Dispensary, and Buguruni CTC. Homes in the wards of districts Ilala, Kinodoni, and Temeke of

Dar es Salaam (Table 1) with reported cholera deaths during August 16, 2015–January 16, 2016, were included in the study.

Case Definitions

A CTC decedent was defined as a patient ≥2 years of age with suspected or confirmed cholera who died following admission to a hospital or CTC during August 16, 2015– January 16, 2016. A community decedent was defined as a person with highly suspected or culture-confirmed cholera who died in the community during August 16, 2015– January 16, 2016.

Case Finding

To obtain names and wards of residence of CTC decedents, we reviewed registers and patient records at hospitals and CTCs. We also abstracted available information on the patient's time of admission, treatment, co-morbidities, and laboratory results. To construct a list of community decedents, we reviewed burial permits of persons who were either dead on arrival to referral hospitals and CTCs or were reported to municipal offices. From the permits, we obtained the decedents' age, sex, residence, date of illness onset, and date of death.

Data Collection

We worked with ward EHOs to obtain the street and administrative leaders of decedents. We requested either the EHO or administrative leader guide us to the home of the decedent. Enumerators interviewed the spouse or caretaker about their knowledge of cholera and water handling behaviors and about the decedent's demographic characteristics, care-seeking behaviors, treatment received (if any), and barriers to care.

The questionnaire was developed in English, translated into Kiswahili, and back-translated into English. Responses were entered onto Galaxy Tablets (Samsung, Seoul, South Korea) into an XLM form by using Open Data Kit software (https://opendatakit.org/), and transferred daily to an encrypted server. Survey results were aggregated into an Epi Info 7 database (https://www.cdc.gov/epiinfo/index.html) for analysis.

Data Analysis

Data was de-identified and analyzed descriptively by using Epi Info version 7 (Centers for Disease Control and Prevention, Atlanta, Georgia, USA). When possible, deaths occurring in CTCs were compared with those occurring in the community. Because data in this convenience sample were not normally distributed, we used the Mann Whitney U test, a nonparametric

method, to compare the 2 groups. These descriptive statistics and nonparametric tests reviewed attributes of persons dying of cholera in CTCs and in the community.

Ethical Consideration

This evaluation was part of the emergency public health response to the cholera epidemic that was ongoing in Tanzania. We limited data collection to a series of cases in Dar es Salaam, and thus, results were not generalizable. To respect confidentiality, we obtained verbal or written informed consent from all respondents. Unique identifiers were protected throughout the course of the investigation and destroyed following data analysis. We performed data analyses only with deidentified data. We obtained ethical clearance through the Muhimbili University of Health and Applied Sciences institutional review board. The US Centers for Disease Control and Prevention institutional review board had determined that this evaluation was not research because the data generated would not be generalizable and would be collected as part of the response to a public health emergency.

Investigation Limitations

This investigation had 2 main limitations. First, because data collection took place >1 month after the date of death, it was possible that accurate recall was a problem for some survey respondents. This risk was mitigated by careful administration of the survey and assistance through prompts when necessary for some questions. In addition, because recall of profound events such as sudden death is often greater than recall of more mundane occurrences, recall bias might not have had a substantial effect. Second, because this investigation was limited to case findings through burial permit data, it is possible that not all community deaths were captured. This might have been especially true earlier in the outbreak when patients who died rapidly from cholera might not have been identified as cholera patients.
