# Supplementary material for: Cholera Mortality during Urban Epidemic, Dar es Salaam, Tanzania, August 16, 2015–January 16, 2016
Source: Emerg Infect Dis. 2017 Dec;23(Suppl 1):S154–7. doi: 10.3201/eid2313.170529 (PMC5711300; doi:10.3201/eid2313.170529)
Supplement: Technical Appendix 2 — Survey given to caregivers of decedents. [file 17-0529-Techapp-s2.pdf]

Article DOI: https://doi.org/10.3201/eid2313.170529

Cholera Mortality during Urban Epidemic, Dar es Salaam, Tanzania, August 16, 2015–January 16, 2016

Technical Appendix 2

MORTALITY INVESTIGATION

Interviewer________________

Date of Interview: ____(day)/____(month)/2015

Address of Deceased:

Region: _________________________________

District: _________________________________

Ward: ____________________________________

Street_____________________________________

Date of death reported on burial permit: ______(day)/__________(month)/2015
Name of the deceased _________________________
DECEDENT UNIQUE ID#___________________
Collect GPS coordinates at end of interview: Latitude______________
Longitude_________________________
Informed Consent (English)

Introduction and Purpose

Hello, I am working with the Tanzanian Ministry of Health and Social Welfare (MOHSW) to investigate the ongoing cholera outbreak. We are trying to understand why people are dying from cholera so we can prevent more people from dying from cholera in the future. Your answers to these questions may help us prevent other people from getting cholera. You and your family are free to choose whether or not to participate in this investigation. You are also free to say no to any part of this investigation. There is no penalty if you or your family do not want to participate.

Procedures

If you decide to participate, we will ask you some questions today. The questions will be about you, your deceased family member, how your family obtained treatment and, if anyone was sick with cholera, details on what happened.

Risks or Discomforts
This study will involve minimal risk to you. Since we will be asking about your deceased family member, some of these questions may make you sad or uncomfortable. You can take a break, skip any questions that are uncomfortable, or end the survey if it is too difficult to talk about your family member.

Benefits
Taking part in the program may help other people because we can learn what communities are doing to deal with the cholera outbreak. The things we learn may prevent other people from dying from cholera in the future and will help to increase support for fighting cholera in communities in Tanzania.

Compensation
There is no cost or payment for being in the study.

Confidentiality
To the extent legally possible, we will keep your answers to all questions secret. All of the information will be password-protected and kept in a special place. We will not put your name or the name of your family members on any report of this project.

Right to Refuse or Withdraw
Your participation today is voluntary. If there are questions you do not like, you do not have to answer them. You can stop at any time after giving your consent. There are no penalties for not being in the study.

Contact information for questions and concerns
If you have questions about the program or feel you have been harmed, you may contact your ten-cell leader or the study coordinator, Amani Massey at 0787754858.

**Q#**	**PRE-INTERVIEW**
**P.0**	Did the interviewee Consent?<br><br>1. Yes 0. No 99. Don’t Know<br><br>***If YES*** ➔**Proceed to P.1**<br>***If NO*** ➔***Thank them and end the interview***
**P.1**	Take a picture of the consent form
**P.2**	Select appropriate enumerator name:<br><br>1. ** 2. *** 3. *** 4. ***
**P.3**	Select District
**P.4**	Select Ward
**P.5**	Select Unique ID of the person Named

Elicit answers from family member of the deceased (if deceased was <16→ ask mother, if ≥16 → ask for head of household/primary caregiver). ***Please use the name of the person who has died wherever it says "deceased".***

Thank you. I would like to start by asking you some questions about your deceased family member and the disease called cholera.

Q#	SCREENING QUESTIONS
**1.**	Have you ever heard of cholera?<br><br>1. Yes 0. No<br><br>If yes → Proceed to Q2<br>If no → Read: "Cholera is a disease caused by a bacteria. Symptoms often include rapid onset watery diarrhea that looks like rice water. People can dehydrate rapidly from this disease."
**2.**	Could you confirm the name of the person who died of rapid onset watery diarrhea (cholera)?<br><br>*Input name into text field.*
**3.**	Our records indicate a date of death on or around {date of death}. Is this correct?<br>1. Yes 0. No 99. Don't know<br><br>If yes → proceed to q5<br>If no → proceed to q4
**4.**	What date did {name} die?<br><br>*Input date into the calendar field.*
**5.**	Did they tell you why {name} died?<br>1. Yes 0. No 77. Decline to answer 99. Don't know<br>If yes → What did they tell you was cause of death? (*Input cause of death into text field)*<br>If no, decline or don't know → Q6
**6.**	Did anyone else in your home die of rapid onset watery diarrhea (cholera) in 2015?<br>1. Yes 0. No<br>If Yes → How many? *Input number in number field*<br>If No → Q7

I am very sorry to hear that. Now I would like to ask you some questions about yourself.

Q#	Respondent Information
**7.**	**Name of Respondent ____**
**8.**	**Age of Respondent ________________________(years)**
**9.**	**Sex of Respondent 1. Male 2. Female**
**10.**	**What is your relationship to the deceased? (***try and interview the spouse or main caregiver of the deceased***)**<br>**1. Spouse**<br>**2. Child**<br>**3. Parent**<br>**4. Sibling**<br>**88. Other (specify)____**
**11.**	**What is the last year of school which you have completed?:**<br>**1. None**<br>**2. Some primary school**<br>**3. Completed primary school**<br>**4. Some secondary school**<br>**5. Completed secondary school or higher**<br>**88. Other (***specify***) ____**

Now I will ask you some questions about *(name of deceased*)

Q#	**Decedent Information**
**12.**	Including (*name of decreased)*, how many people lived in the house (slept here and shared meals) at the time he/she died?<br><br>__________ *(number of persons, including deceased)*
**13.**	What was the age of {name of deceased}?<br>________________________
**14.**	What was the sex of (name of deceased)?<br>1. Male 0. Female
**15.**	What was the religion of (name of deceased)?<br>1. Christian<br>2. Muslim<br>88. Other(specify)_______________________<br>99. Don't know
**16.**	What was the last year of school *(name of decreased)* completed? *(CIRCLE ONE)*<br>1. None<br>2. Some primary school<br>3. Completed primary school<br>4. Some secondary school<br>5. Secondary school or higher<br>88. Other (*specify*) ____
**17.**	What was the occupation of *(name of decreased)?*<br>1. Unemployed<br>2. Housewife<br>3. Petty trader<br>4. Food vendor<br>5. Healthcare worker<br>6. Day worker<br>7. Student<br>88. Other ____<br>99. Unknown
**18.**	Do your household own any of the following? *(ASK EACH OPTION)*

	**1. Yes**	**0. No**	**99. Don't know**
Radio	❒	❒	❒
Bank Account	❒	❒	❒
Iron (Charcoal or electric)	❒	❒	❒
Motorcycle	❒	❒	❒
Mobile telephone	❒	❒	❒
Animals (i.e. cows, goats, chickens, pigs)	❒	❒	❒
Television	❒	❒	❒

Q#	
**19.**	What type of fuel does your household mainly use for cooking?<br>1. Charcoal 2. Firewood 88. Other ________________ 99. Don't know
**20.**	What is the main source of energy for lighting in the household?<br>1. Electricity 88. Other _______________ 99. Don't know

Now I will ask you about the **symptoms** that (*name of deceased*) had during the week prior to dying

Q#	Symptom Information
**21.**	What symptoms did {name of deceased} have in the week before dying?<br>Vomiting 1. Yes 0. No 99. Don't Know<br>Diarrhea 1. Yes 0. No 99. Don't Know<br>Fever 1. Yes 0. No 99. Don't Know<br>Headache 1. Yes 0. No 99. Don't Know<br>Other ________________________________
**22.**	Do you know what date {name of deceased}'s diarrhea began?<br>1. Yes 0. No<br><br>If yes → *Fill out date in calendar*<br>If no → Q23
**23.**	On average, how many stools did *(name of decreased)* have after he/she became ill?<br>1. Less than 3 stools per day<br>2. Between 3-10 stools per day<br>3. Greater than 10 stools per day<br>99. Don't know
**24.**	Approximately how many hours after developing diarrhea did the deceased die?<br>1. Less than 4 hours<br>2. 5-8 Hours<br>3. 9-12 Hours<br>4. 13-24 Hours<br>5. 25-48 Hours<br>6. Greater than 48 hours<br>99. Don't know/don't remember
**25.**	*[If deceased was older than 11 and female, ask this question]* Was (name of *deceased*) pregnant?<br>1. Yes 0. No 99. Don't Know
**26.**	Was the (*name of deceased*) ever told by a health care provider that he/she had any of the following: (*READ EACH CHOICE)*<br>Attention: Ask these questions in a private manner.<br>Tuberculosis Yes No Don't Know<br>Cancer Yes No Don't Know<br>HIV/AIDS Yes No Don't Know<br>Is there any other previous illness that I did not ask about?<br>Other illness<br>____

We would also like to understand more about the usual health and diet of (*name of deceased*).

**27.**	***[If deceased is less than 15 years of age, SKIP to Q30]***<br>Did the deceased ever drink alcohol (beer, local brew, Mnazi/Chibuku)?<br>1. Yes 0. No 99. Don't Know<br><br>***If YES → Q28***<br>***If NO → Q30***
**28.**	Did the deceased drink alcohol every day?<br>1. Yes 0. No 99. Don't Know
**29.**	Did the deceased drink alcohol the day he or she became ill?<br>1. Yes 0. No 99. Don't Know

Now I will ask you about the care (name of deceased) received **at home** after becoming ill with cholera.

Q#	Care at Home
**30.**	Did (*name of deceased*) take any medicine or treatment at home after this illness began?<br>1. Yes 0. No 99. Don't know<br><br>***If YES → Q31***<br>***If NO or Don't know → Q32***
**31.**	What medicines did he/she take at home? (***Do NOT READ,*** *CHECK ALL THAT APPLY*)<br>1. Antibitoic<br>Specify antibiotic (if caregiver knows)____<br>2. ORS<br>3. Traditional medicines/herbs<br>88. Other (*specify*) ____________________________<br>99. Don't know
**32.**	Did (*name of deceased*) take ORS at home?<br>1. Yes 0. No 99. Don't know<br>***If YES → Q33***<br>***If NO → Q34***<br>***If don't know → Q35***
**33.**	Where was the ORS obtained? *(CIRCLE ONE)*<br>1. Health Center (Dispensary)<br>2. Market<br>3. Pharmacy<br>4. From a petty trader<br>5. From a friend/neighbor/family member<br>6. Red Cross<br>7. Another NGO<br>88. Other (*please specify):-*<br>____<br>99. Don't know

**34.**	Why didn't (name of deceased) take ORS at home? (DO NOT READ, circle all that apply)<br>1. He/she did not know what ORS was<br>2. ORS was too expensive<br>3. ORS was not available in the local stores<br>4. He/she did not know where to find ORS<br>5. He/she did not think ORS would help the symptoms of this illness<br>88. Other (*please specify):-*<br>____<br>99. Don't know
**35.**	Did the deceased drink fluids <u>at home</u> after the illness began?<br>1. Yes 0. No 99. Don't know<br><br>***If YES→ Q36***<br>***If NO or Don't know →Q37***
**36.**	What did they drink? *(CIRCLE ALL THAT APPLY)***READ ALL OPTIONS**<br>1. Water<br>2. Juice/soda/soft drink<br>3. Home-made Water/sugar/salt solution<br>4. Traditional medicine/herbs<br>88. Other ____<br>99. Don't Know

Now I will ask you about the care (*name of deceased*) received outside the home **after** becoming ill with cholera. This could be at a hospital, dispensary, or care given by a traditional healer.

**Q#**	**Outside Care**
**37.**	Once your family member became ill, how long did it take for he or she to seek care?<br><br>________________ Hours or Days (circle one)
**38.**	Did the (*name of deceased*), or a relative acting on their behalf, seek any type of care outside the home (such as at a health center/hospital/CTC) after this illness began?<br>1. Yes 0. No 99. Don't know<br><br>***If YES→ Q41***<br>***If NO → Q39***

<table>
<tr><td>39.</td><td>What is the main reason (name of deceased) did NOT go to a hospital or health center for treatment?
(DO NOT READ. CIRCLE ONLY <u>ONE</u>)
1. Died in transit/on the way to the health center
2. Cost of care at the health center was too much
3. Did not have transportation
4. Did not feel safe travelling at night
5. Transportation cost too much
6. Clinic was too far
7. Too ill to leave home
8. Did not know s/he had cholera
9. Did not know that cholera could be treated
10. Did not want others to know that s/he was sick (shame)
11. Did not think s/he needed medical care
12. Thought God would cure them
13. Prefered a traditional healer
14. Facility was closed
15. Too long of a waiting time at health center
16. Fear of mistreatment by the medical staff
88. Other________________________________
99. Don't Know</td></tr>
<tr><td>40.</td><td>Are there any other reasons that he/she didn't seek care at the hospital or health center?
(DO NOT READ. CIRCLE ALL THAT APPLY)
1. Died in transit/on the way to the health center
2. Cost of care at the health center was too much
3. Did not have transportation
4. Did not feel safe travelling at night
5. Transportation cost too much
6. Clinic was too far
7. Too ill to leave home
8. Did not know s/he had cholera
9. Did not know that cholera could be treated
10. Did not want others to know that s/he was sick (shame)
11. Did not think s/he needed medical care
12. Thought God would cure them
13. Prefered a traditional healer
14. Facility was closed
15. Too long of a waiting time at health center
16. Fear of mistreatment by the medical staff
88. Other________________________________
99. Don't Know</td></tr>
</table>

Where did (name of deceased) seek care? *[read choices, check all that apply, travel time to facility in hours/minutes, how long they waited at the treatment facility, record if they spent the night and if they were discharged alive)*

Circle all that apply	Primary Mode of Transport used<br>1. Taxi<br>2. Bajaji<br>3. Walked<br>4. Boda boda<br>5. Dala dala<br>6. Carried by family/ friends<br>7. Ambulance<br>8. Other	How long did it take {name deceased} or the relative to get from home to treatment location? (*integer)*<br>_____<br>Hrs / Minutes (*circle*)	Waiting time at the location/ health facility<br>1. Immediate care/no wait<br>2. <1 hour<br>3. 1 – 3 hours<br>4. >3 hours	Treatment received? *(CIRCLE)*	Spend the night? *(CIRCLE)*	Discharged Alive? *(CIRCLE)*
**42.** Pharmacy<br>1.Yes<br>2. No<br>99. DK<br>(name)_____<br>_____<br>Who sought care?<br>1. Relative<br>2. Deceased		Hrs / Minutes		1. ORS<br>2. IV fluids<br>3. Antibiotics<br>4. Other:<br>____________	Not applicable	1.Yes<br>2. No<br>99. DK
**43.** Traditional healer<br>1.Yes<br>2. No<br>99. DK<br>(name)_____<br>_____<br>Who sought care?<br>1. Relative<br>2. Deceased		Hrs / Minutes		1. ORS<br>2. IV fluids<br>3. Antibiotics<br>4. Other:<br>____________	1.Yes<br>2. No<br>99. DK	1.Yes<br>2. No<br>99. DK
**44.** Dispensary (name)<br>__________<br>1.Yes		Hrs / Minutes		1. ORS<br>2. IV fluids<br>3. Antibiotics<br>4. Other:	1.Yes<br>2. No<br>99. DK	1.Yes<br>2. No<br>99. DK

		2. No<br>99. DK				___________			
		**45.** Hospital Name: ___________<br>1.Yes<br>2. No<br>99. DK		Hrs / Minutes		1. ORS<br>2. IV fluids<br>3. Antibiotics<br>4. Other: ___________	1.Yes<br>2. No<br>99. DK	1.Yes<br>2. No<br>99. DK	
		**46.** CTC/Transit Center Name:___________<br>1.Yes<br>2. No<br>99. DK		Hrs / Minutes		1. ORS<br>2. IV fluids<br>3. Antibiotics<br>4. Other: ___________	1.Yes<br>2. No<br>99. DK	1.Yes<br>2. No<br>99. DK	
		**47.** Other (specify): ___________		Hrs / Minutes		1. ORS<br>2. IV fluids<br>3. Antibiotics<br>4. Other: ___________	1.Yes<br>2. No<br>99. DK	1.Yes<br>2. No<br>99. DK	

Now I will ask some questions about cholera in general.

Q#	Knowledge
**48.**	Please name all the ways you received information about cholera? ***(Do not read**, CIRCLE ALL THAT APPLY)*<br>1. Radio<br>2. Television<br>3. Newspaper<br>4. Flyer/brochure/poster<br>5. Community health worker<br>6. Community meeting<br>7. Street chairperson<br>8. 10 cell leader<br>9. Health care facility staff<br>10. Friend<br>11. Family member<br>12. Public dances<br>13. Students from the local schools<br>88. Other (specify) _____________<br>99. Don't know/don't remember
**49.**	Can cholera be prevented?<br>1. Yes 2. No 99. Don't know<br>If Yes →50<br>If No → 51
**50.**	*If yes,* Please name all the ways to **prevent** cholera (***DO NOT READ**, CIRCLE ALL THAT APPLY)*<br>1. Boil or treat water with chlorine<br>2. Build and use latrines<br>3. Wash hands with soap and water (ash or sand)<br>4. Cook food well<br>5. Cover food to protect from flies<br>6. Wash vegetables and fruit<br>7. Clean house with chlorine solution<br>8. Seek treatment for watery and/or bloody diarrhea<br>9. Take an antibiotic pill<br>10. Cholera cannot be prevented<br>11. Not touching someone with cholera<br>12. Avoid sharing food<br>13. Avoid gatherings<br>14. Vaccine<br>88. Other (specify)_________________________<br>99. Don't know
**51.**	Do you think cholera can be treated?<br>1. Yes 0. No 99. Don't Know

**52.**	Please name all the things you would do if you were suffering from cholera. (***DO NOT READ***, *CIRCLE ALL THAT APPLY)*<br>1. Drink oral rehydration solution/ ORS<br>2. Go to a pharmacy and buy antibiotics<br>3. Go to a health facility<br>4. Go to a cholera treatment center<br>5. Go to a traditional healer<br>88. Other (specify)________________________<br>99. Don't know
**53.**	Do you think it is necessary to go to a medical facility, like a health center, clinic, or hospital, if you have cholera?<br>1. Yes 0. No 99. Don't know<br><br>***If YES→ Q54***<br>***If NO/Don't know → Q55***
**54.**	When should you go to a medical facility if you think you have cholera? ***(Do not read,*** *CIRCLE ONE)*<br>1. Immediately<br>2. After 24 hours (1 day)<br>3. If there is vomiting and you cannot drink ORS or fluids<br>4. If there is blood in the stool<br>5. If diarrhea does not stop<br>88. Other (*specify*)<br>____<br>99. Don't know

Now, I will ask you some questions about your drinking water.

**Q#**	**Water Information**
**55.**	Did your household treat your drinking water before (*name of deceased*) became ill with cholera?<br>1. Yes 0. No 99. Don't know<br><br>***If YES → Q56***<br>***If NO/Don't know → Q57***
**56.**	What treatment method(s) did you use? *(CIRCLE ALL THAT APPLY)*<br>1. Boil<br>2. jik/chlorine<br>3. Aquatabs<br>4. Waterguard<br>5. Filter<br>88. Other (specify) ______________________<br>99. Don't Know

**57.**	Did you treat your drinking water today?<br>1. Yes 0. No 99. Don't know<br><br>***If YES → Q58***<br>***If NO/Don't know → Q59***
**58.**	What treatment method(s) did you use? *(CIRCLE ALL THAT APPLY)*<br>1. Boil<br>2. jik/chlorine<br>3. Aquatabs<br>4. Waterguard<br>5. Filter<br>88. Other (specify) ________________________<br>99. Don't Know
59.	Did you collect your drinking water for the household from a DAWASCO source?<br>1. Yes 0. No 99. Don't know<br><br>**If yes → Q60**<br>**If No or Don't know → Q61**

	**60.** Select all that apply:<br>DAWASCO SOURCE<br>1. Open deep well<br>2. Protected or covered deep well<br>3. Borehole<br>4. Piped water to house<br>5. Community tap/kiosk<br>6. Water tank<br>7. Water bowzer/truck<br>8. Water vendor (jerrycan)<br>9. Bottled water<br>10. Other (specify)_________________	61. Select all that apply:<br>NON-DAWASCO SOURCE<br>1. Open deep well<br>2. Protected or covered deep well<br>3. Shallow well/hand-dug well<br>4. Lake/Pond/River/Stream<br>5. Borehole<br>6. Rain water catchment<br>7. Piped water to house<br>8. Community tap/kiosk<br>9. Water tank<br>10. Water bowzer/truck<br>11. Water vendor (jerrycan)<br>12. Bottled water<br>13. Other (specify)_________________

**62.**	"Do you have any products used for water treatment in the house?"<br>1. Yes 0. No<br>***If YES → Q63***<br>***If NO → Q64***

**63.**	*If YES, "*MAY I SEE THE PRODUCT?" *(CIRCLE ALL THAT APPLY)*<br>1. jik/chlorine<br>2. Aquatabs<br>3. Waterguard liquid/powder<br>4. Filter<br>88. Other (specify) ________________________
**64.**	"Do you have ORS in house?" (PLEASE OBSERVE DIRECTLY)<br>1. Yes 0. No
**65.**	Do you know how to make oral rehydration salts?<br>1. Yes 0. No<br><br>**If yes → Q66**<br>**If no → Q67**
**66**.	"Please tell me how you prepare ORS."<br>(ANSWER: Take 1L of water into a container, <u>treat the water</u>, and stir-in the ORS packet)<br><br>0. Do not know how to correctly make ORS<br>1. Correctly identify steps to make ORS
**67.**	**Would you be willing to speak with some of our team and other families that also had relatives die from cholera? (Do not read, but *this is for people that would be interested in focus groups)***<br><br>0. No<br>1. Yes → Take the name and phone number of the relative<br>____

Make the below quick observations regarding the home. If the interview is taking place outside the home, please ask the interviewee the following questions and read each option out loud.

**Q#**	***HOUSEHOLD OBSERVATIONS***
**68.**	*What type of roofing does this household have (CIRCLE ONE)(OBSERVATION ONLY)*<br>1. Thatch<br>2. Metal/iron sheet<br>3. Tile<br>4. Wood<br>5. Cement<br>88. Other: _____________________________________
**69.**	*What type of flooring does this household have (CIRCLE ONE) (OBSERVATION ONLY)*<br>1. Mud<br>2. Wood<br>3. Cement<br>4. Tile/linoleum<br>88. Other: ____

**70.**	*What type of material is used for the walls (CIRCLE ONE) (OBSERVATION ONLY)*<br>1. Mud<br>2. Metal<br>3. Wood<br>4. Cement/plaster<br>5. Bricks/blocks/stones<br>88. Other: ______________________________

Thank you very much for your participation. We are very sorry for the loss of (*name of decedent)*

COLLECT GPS COORDINATES OF THE HOME FOR THIS SURVEY AT ITS CONCLUSION.

The findings and conclusions in this report are those of the authors and do not necessarily represent the official position of CDC.
